# XML schemas for common bioinformatic data types and their application in workflow systems

**DOI:** 10.1186/1471-2105-7-490

**Published:** 2006-11-06

**Authors:** Philipp N Seibel, Jan Krüger, Sven Hartmeier, Knut Schwarzer, Kai Löwenthal, Henning Mersch, Thomas Dandekar, Robert Giegerich

**Affiliations:** 1 Department of Bioinformatics, Biocenter, University of Würzburg, Würzburg, Germany; 2 Bioinformatics Group, Practical Computer Science Department, Faculty of Technology, Bielefeld University, Bielefeld, Germany; 3 Department of Bioinformatics, UKG, University of Göttingen, Göttingen, Germany; 4 Distributed Systems and Grid Computing, Central Institute for Applied Mathematics, Research Centre Jülich, Jülich, Germany

## Abstract

**Background:**

Today, there is a growing need in bioinformatics to combine available software tools into chains, thus building complex applications from existing single-task tools. To create such workflows, the tools involved have to be able to work with each other's data – therefore, a common set of well-defined data formats is needed. Unfortunately, current bioinformatic tools use a great variety of heterogeneous formats.

**Results:**

Acknowledging the need for common formats, the Helmholtz Open BioInformatics Technology network (HOBIT) identified several basic data types used in bioinformatics and developed appropriate format descriptions, formally defined by XML schemas, and incorporated them in a Java library (BioDOM). These schemas currently cover sequence, sequence alignment, RNA secondary structure and RNA secondary structure alignment formats in a form that is independent of any specific program, thus enabling seamless interoperation of different tools. All XML formats are available at , the BioDOM library can be obtained at .

**Conclusion:**

The HOBIT XML schemas and the BioDOM library simplify adding XML support to newly created and existing bioinformatic tools, enabling these tools to interoperate seamlessly in workflow scenarios.

## Background

Today, bioinformatic analyses are becoming more and more complex tasks. Large scale analyses usually integrate several independently calculated results. A good example for such a complex application is automated genome annotation, a process starting from detecting promotor regions and automatically predicting genes to complete functional annotation of novel genomes [1-3]. To accomplish such complex tasks, many different tools are needed. This can lead to problems when trying to find a simple way to combine the necessary tools.

Workflow tools, such as Taverna [4], Wildfire [5] or Pegasys [6], address these problems and provide a user interface to orchestrate bioinformatic tools into complex workflows. These workflow systems mostly require that the programs to be integrated are provided as webservices [7] or need extra programming effort to make the tools interoperate with each other [8].

Webservices provide a well defined programming interface to integrate tools into applications over the internet or other network connections. Currently a growing number of bioinformatic applications are provided as webservices, such as Eclair [9], SOAP-based services provided by the EBI [10], Biosphere [11], AliasServer [12], Soap-HT-BLAST [13], biological SOAP servers and webservices provided by the public sequence data bank [14], INCLUSive [15], and BioMoby [16]. Therefore, using webservices to build complex networked toolchains is a widely accepted solution.

To be able to create workflows consisting of webservices, the involved tools need to work correctly with each other's data – therefore, a common set of well-defined data formats is necessary. Furthermore, it should be possible to validate the correctness of data given in these formats, enabling the system to inform the user of problems caused by the data as early as possible. Unfortunately, current bioinformatic tools use a great variety of heterogeneous formats for reading data and storing their results. Some of these formats, like FASTA [17] for sequence data, CLUSTAL [18] for alignments or Vienna style DotBracket [19] for RNA secondary structure information, were originally designed to be read in a standard text editor. These formats often lack consistency: There are, for example, many different interpretations of the 'correct' FASTA file format. A formal, machine-readable definition of the formats is often missing. For instance, some applications use lower- and uppercase characters in the raw sequence to encode additional annotations in FASTA, while others append extensive information to the header line. Various tools often use the same encoding to store different information. Because of these circumstances automated processing of FASTA can be difficult or even impossible. Of course FASTA isn't designed for this kind of additional annotations, but this fact doesn't prevent people from using it for other purposes, e.g. to store binding site information.

To overcome the problem of identifying, specifying and extracting the desired information, the use of XML [20] for all kinds of bioinformatic data provides a good solution. XML is platform and programming language independent and can be formally defined using the XML Schema language [21]. Modern webservice technology is based on XML [22] and the data exchanged by these webservices also relies on XML (a well known example is the eUtils webservice at NCBI [23,24]). Therefore, well specified XML data seems to be a useful, modern and accepted technology for exchange of bioinformatic data.

Today, a simple search on the WWW reveals literally dozens of existing XML formats for bioinformatical data, and several sites collecting information about these formats. A well-known example is the XML web site of Paul Gordon [25], which contains links to various XML information sites relevant for molecular biology. But despite the multitude of possible formats, compact and simple data formats for frequently used simple information such as *only *sequence or alignment data are still missing, as the existing formats are usually quite complex or suffer from other limitations as discussed below.

Furthermore, most of the existing XML formats are defined by XML Document Type Definitions (DTDs) only, but DTDs do not allow specification of conditions and constraints on the content of XML tags. This makes syntactic validation of the actual data described by such formats, e.g sequence data, impossible. Fortunately, a more modern approach is available by usage of XML Schema Definitions (XSD) [21] instead of DTDs, which allow a much more rigid definition of XML syntax. Unfortunately, only very few existing XML formats use this approach, and most of those which do, do not use XSDs potential properly.

An overview of the most prevalent existing XML formats for sequence based bioinformatical data (which was the initial focus of our work) and a short evaluation for determining their general suitability as data formats in workflow scenarios is given in table 1. As can be seen, most current formats in this area do have certain shortcomings. These cause problems when trying to use them within a workflow application scenario.

**Table 1:** Commonly used XML formats and their features

**Name**	**Scope**	**Pro**	**Contra**
AGAVE	sequence/annotation	XML schema available, stable, format is open and seems to be actively maintained, well documented	XML schema is in BETA status (since Feb. 2003), XML schema defines no namespace, no restriction of sequence data
BioML	sequence/annotation	-	no XML schema available (DTD only), unclear if it is stable and maintained (last modified 1999)
BioSeq	plugin of readseq	-	no XML schema available (DTD can be generated), maintenance and stability unclear, undocumented
BSML	sequence/annotation, sequence alignments	well documented	no XML schema available (DTD only), unclear if it is maintained any longer (last updated 2002)
chadoXML	data base format	-	no XML schema available (DTD can be generated), part of the GMOD XORT software package, undocumented
EMBLxml	sequence data base format	XML schema available	XML schema defines no namespace, no restriction on content elements
GAMEXML	sequence/annotion	used in different OS projects, seem to be stable	no XML schema available (DTD only), maintenance unclear
INSDseq	sequence data base format	lightweight	no XML schema available (DTD only)
MSAML	sequence alignments	-	no XML schema available, project page unreachable (DTD on third party page), maintenance unclear
RNAML	RNA sequence, structure and experimental data	XML schema available, well documented	XML schema defines no namespace, complex and unmanageable, license and maintenance unclear (last modified 2002)
TinySeq	sequence data	stable, active, lightweight	no XML schema available (DTD only), undocumented

The list above contains a summary evaluation of formats with the same scope of application as the HOBIT formats. A more complete list (including detailed features) is available at [57].

For other application areas like protein interaction, microarray experiments, phylogenetics and systems biology, other formats like e.g. PSI-ML [26], ProML [27], Mage-ML [28], PhyloXML [29] and SBML [30] exist, which have been adopted in varying degrees by their respective communities. These applications are not yet covered by our project.

## Implementation

### Basic Concepts

Due to the need for common data formats, we have identified several basic data types used throughout the bioinformatic community and created elementary format descriptions (see table 2), formally specified by XML schemas, and a library (BioDOM) to create XML files according to these schemas and additionally convert from prevalent formats to the XML formats and vice versa. These XML formats are extensively used within the HOBIT project to facilitate interoperation between the bioinformatic webservices provided by the project members at several different universities and research institutes throughout Germany. Nevertheless, we would especially like to emphasise the fact that although the formats have initially been defined within the HOBIT project, their use is by no means restricted to this context. On the contrary, they have been explicitly designed to be useful building blocks for any user in the bioinformatic community, and their usage for data exchange between bioinformatic tools is highly encouraged. In the following, we describe some of these XML schemas and show examples of their application.

#### XML schema structure

Some implementation guidelines were defined for the different HOBIT XML schemas to guarantee consistency in development and results. These guidelines are as follows:

XML schemas grant the ability to validate the payload data, which is not the case in DTDs. Since this ability is important in workflow environments, XML Schema based format definitions are a requirement. Another requirement originating from the distributed workflow scenario is stability. Therefore, only stable specifications can be used. In accordance with the HOBIT guidelines it is mandatory that the format is not bound by a closed licence restriction, but may be used and extended freely.

Active maintenance of formats is also essential, since this is especially important in an area of rapid development like bioinformatics. Likewise it should be possible to extend the format to accomodate special use cases.

Two additional features of formats that we recommend, but not require, are simplicity and usage of building blocks. Both features improve the usability of the format.

**Figure 1:**
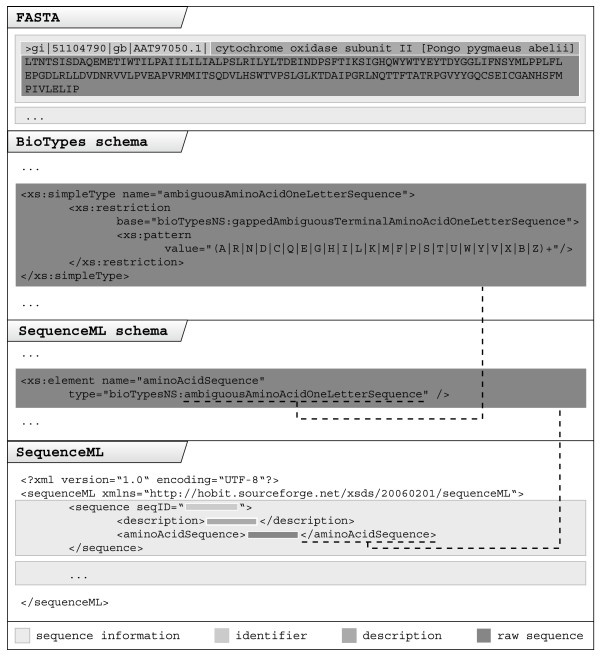
**Basic concept of HOBIT XML schemas**. The basic concept of HOBIT XML schemas is explained step by step using SequenceML as an example. First an amino acid sequence with id and description in the well known FASTA format is converted to SequenceML. The color coding highlights the transformed content. In SequenceML it is possible to differentiate between various sequence types (in this case an amino acid sequence), defined by the SequenceML schema. The SequenceML schema derives its basic type information from BioTypes.

We do not necessarily want to replace existing schemas. A new schema was developed only if no available schema was suitable for the given requirements. All the schemas make extensive use of inheritance. HOBIT XML format descriptions are based on two XML schemas, containing elementary types: Basic biological types like amino acid sequences are defined in a collection named BioTypes [31]. The usage of this types in the newly developed XML schemas is illustrated in figure 1. More technical elements, e.g. parameters of a commandline application, often needed in the XML schemas, are collected in the HobitTypes [32]. To allow widespread and diversified use of the schemas, some extension points were incorporated into the schemas. Information unaccounted for in a given version of a schema can easily be added as attribute. This is accomplished by adding XSD anyAttribute declarations to central tags.

Since this opens a possibility for improper extension of a given schema, but reasonable extensions should be considered during validation, a mechanism to support ongoing development was necessary. To fulfill this requirement, a public Wiki page was installed [33]. Every interested person is invited to make suggestions for the improvement of the schemas directly in the Wiki, working cooperatively with other persons to improve the schema definitions. The XML schemas can be obtained from the subversion repository located at [34]. For quality control purposes, changes can be commited only by registered SourceForge [35] BioSchema project [36] members.

### Sequence formats

#### SequenceML

SequenceML deals with all kinds of simple sequence information often used as input for several common bioinformatic tools. It is designed to be used as an XML replacement of the FASTA [17] format, containing all of FASTA's information while avoiding that format's aforementioned consistency problems. SequenceML differentiates between nucleic and amino acid sequences following the IUPAC standard and also allows the user to add free sequence information based on basic types defined by BioTypes [31] (figure 1). SequenceML also supports a mandatory sequence id and an optional detailed sequence description. SequenceML does not contain any annotation information.

#### SequenceAnnotationML

SequenceAnnotationML is based on SequenceML. While SequenceML contains raw sequence information, SequenceAnnotationML allows additional annotations. Thus, while SequenceML is often used as input for bioinformatic tools, SequenceAnnotationML can be used to store the result. SequenceAnnotationML allows modelling sites of interest of small sequences (DNA, RNA or protein). Furthermore it is possible to encapsulate whole genome annotations due to its recursive structure.

#### AlignmentML

AlignmentML is a format describing (multiple) alignment information any alignment program like CLUSTALW [18], DCA [37] and Dialign [38] can produce. Similar to SequenceML, different sequence types are supported.

### RNA secondary structure formats

#### RNAStructML

RNAStructML is a format for storing RNA secondary structure information. The most widely used application for RNA tools, such as RNAshapes [39], RNAfold [19] and Mfold [40] is the proprosal of RNA secondary structures, based on thermodynamic principles. RNAStructML is inspired by SequenceML and uses Vienna style DotBracket strings for storing information about RNA secondary structures.

#### RNAStructAlignmentML

RNAStructAlignmentML is a format for storing RNA secondary structure alignments as computed by e.g. RNAforester [41] or RNAalifold [42]. RNAStructAlignmentML uses an RNAStructML-like architecture, but is based on AlignmentML instead of SequenceML.

### BioDOM

To simplify the usage of the HOBIT XML formats, an easy-to-use Java library (BioDOM) has been developed. BioDOM provides an easy way to build XML files following the HOBIT format descriptions from inside the user's own programs. It is designed to be a modular system which can easily be extended as necessary to accomodate new formats. Additionally, BioDOM provides functions to convert native non-XML output of various bioinformatic tools to the HOBIT XML formats.

The BioDOM library contains one Java class for each natively supported XML format, which implements methods to create the corresponding data structure by adding the necessary parts to the new document or importing data from ordinary data formats to XML elements.

Each of these classes is based on the abstract class AbstractBioDOM, which provides commonly required methods for all converters, e.g. for setting and getting the documents object model (DOM) content, validating the document against an XML schema or creating a string representation of the XML data contained in the object. AbstractBioDOM also provides a general mechanism for XML-to-XML format conversion via XSLT [43] scripts. Finally, some methods for accessing the logging and error/exception handling facilities of the BioDOM library are integrated. This allows for graceful degradation of the system and user notification in case of erroneous input data or unforeseen circumstances during data creation or conversion.

The current version 1.2 of BioDOM supports the HOBIT XML formats SequenceML, AlignmentML, RNAStructML and RNAStructAlignmentML, allowing creation of documents in these formats and, additionally, conversion from and to (multiple) FASTA, CLUSTALW and the Vienna style DotBracket format. XSLT converters for TinySeq [44], INSDseq [45] and EMBLxml [45] are also provided. Owing to its modular design, BioDOM can very easily be extended by third party XSL scripts or own Java classes. Furthermore it is under constant development and testing to support additional data formats.

## Results & Discussion

### HOBIT XML formats

Recent approaches like RNAML [46] or PDBML [47] try to model all possible aspects of a complete field of application in one single XML schema. In the case of RNA this means storing sequences, secondary structures, tertiary structures and even all kinds of experimental data in only one file.

Most programs focus on a specific application, and in this case storing all data leads to huge and unmanageable XML documents, compare e.g. the size of NCBI ASN.1 formatted GenBank data versus the same data's XML representation, which can be about one order of magnitude (or even more) bigger. While keeping the whole complex data in one file might be appropriate for archiving, data exchange in distributed workflow scenarios requires the information to be available in a compact format to allow short response times. This compact format can be achieved in two ways. One is to declare many parts in a complex XML schema 'optional' to reduce the overhead for the different usage scenarios, the other is to use more simple schemas representing small and simple building blocks. The HOBIT XML schemas are designed following the building blocks concept, which is, from our point of view, the preferred solution for the application in workflow systems. Our rationale for this is as follows:

While it is of course possible to extract the data from larger XML data structures using XSLT or XPath expressions, it is much more difficult to generate workflows from webservice modules exchanging general XML data. The extraction patterns have to be defined either by the webservice developer or the person who builds the workflow. In the first case, the webservice is called with an XML file conforming to the complex XML schema as input, but the input doesn't explicitly represent the data the webservice really requires. The second case would require the person building the workflow to have the knowledge to define the extraction patterns for every connection between webservices to handle the in- and output. By using small XML schemas, only the data really used by the webservice is modelled. This makes the handling much more intuitive for the typical non-XML-savvy user of a workflow system.

Additionally, declaring many parts as 'optional' in larger XML schemas leads to a much more complex validation process, e.g. if the XML content elements a webservice requires as input are declared as 'optional' in the XML schema of the previous output data, the service can detect the existence of the required data *only at runtime*.

To illustrate the problems of using general XML schemas in workflow systems, consider a simple workflow for predicting and drawing the secondary structure of a given RNA sequence. The workflow consists of two webservices, the first one a service for predicting the secondary structure and the second one a service that creates a picture from the prediction afterwards. In the case of general XML schemas, like RNAML [46], the first webservice would require XML input data conforming to the general schema and produce XML output data conforming to the same general schema, but adding the RNA secondary structure information. Both XMLs can be validated with the same complex schema, but it is not known whether the output really contains the required data until the second webservice is called. In the case of using building blocks the first service requires SequenceML as input and produces RNAStructML as output, the second service requires RNAStructML as input and generates an image as output. Due to these input/output requirements, the webservices can only be connected if the output of the first service contains the data the second service requires, and this enables the system to validate the correctness of the connections between single services during the construction process of the workflow. Therefore, possible type errors can be detected and prevented *before *a (possibly very expensive or time-consuming) actual run of the workflow. (Of course, errors due to problematic data produced during workflow execution are still possible and can in principle not be prevented.)

Another problem not directly adressed by the HOBIT XML formats themselves is the task of finding and selecting the relevant information from existing XML archive formats to acquire only the data needed for a specific application. After this task has been accomplished, the lightweight HOBIT formats can then be used for further transfer of the data inside a workflow. There are several approaches to solving the data selection problem. Within the BioDOM framework, XSL scripts can be used to select and transform the desired information. If another solution already exists for a specific use case (like e.g. [8]), it can easily be combined with our work.

The HOBIT XML schemas are open for extensions and especially new formats. An open community environment based on a Wiki system is provided to allow discussion and rapid development [48]. For collaborative XML schema enhancements, all XML schemas are available at a subversion repository [34]. By providing open access to our XML schemas, we try to address the common problem of data format extension, e.g. adding features to a existing data format vs. having to define a new one.

### Easy integration

We have implemented a Java package (BioDOM), which can be used to build the supported XML formats directly from an application's internal data structures. BioDOM can also convert between different XML formats (HOBIT defined and others) using XSLT. In addition to these functions, there are conversion functions for many commonly used non-XML formats, which allow traditional tools and services a smooth transition from their data formats towards the XML formats presented in this study.

**Figure 3:**
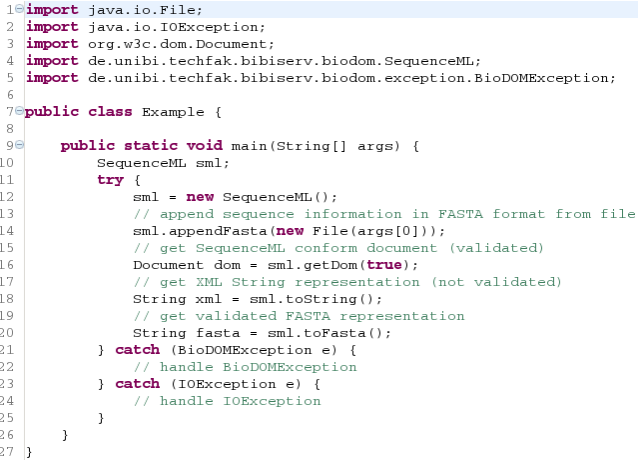
**Example of BioDOM integration**. This code sample shows exemplarily how to use the SequenceML API of BioDOM. First an empty Se-quenceML object is generated (line 12), afterwards a FASTA formatted file is appended (line 14). Some of the possibilities for further processing, as shown in the comments, are given in lines 16 to 20.

Figure 3 shows a typical use case for integrating BioDOM as a library. In addition to this, BioDOM can also be used online to convert bioinformatic data automatically by using the BioDOM webservice [49]. This is especially useful for current workflow systems to connect webservices with incompatible, but convertable data, without any programming effort. For testing purposes and small amounts of data, the conversion functions of BioDOM can additionally be used manually by using the online submission form [49].

For now, BioDOM is focused primarily on providing support for creation of data in the HOBIT XML formats plus simple conversion routines from existing well-known non-XML formats. It is intended to add interfaces to BioJava [50] objects and methods in later versions. Implementations of the BioDOM API in other programming languages are also planned.

### SOAP based webservices

Table 3 shows a list of available webservices supporting HOBIT XML formats as input and ouput. Currently (June 2006), ten webservices from different bioinformatic fields are available. A constantly updated list of offered webservices can be obtained from the HOBIT website [51].

**Table 3:** Available webservices supporting HOBIT XML formats

**Name**	**Description**	**Supported formats (input/output)**
CLUSTALW [18]	general purpose multiple sequence alignment program	SequenceML/AlignmentML
DCA [37]	divide-and-conquer multiple sequence alignment program	SequenceML/AlignmentML
Dialign [38]	computation of an alignment based on segment-to-segment comparison	SequenceML/AlignmentML
E2G [52]	aligning genomic sequence to cDNA and EST data sets	SequenceML/EBIApplicationResult
ITS2 [58-60]	database of more than 20,000 rRNA internal transcribed spacer 2 (ITS2) secondary structures revealed by homology modeling	-/RNAStructML
pknotsRG [61]	RNA secondary structures folding, including the class of simple recursive pseudoknots	SequenceML/RNAStructML
realsplice	information about regulated alternative spliced genes	-/SequenceAnnotationML
REPuter [62]	computes all maximal duplications and reverse, complemented and reverse complemented repeats in a nucleic acid sequence	SequenceML/EBIApplicationResult
RNAshapes [39]	computation of a small set of representative structures of different shapes, computation of shape probabilities, and comparative prediction of consensus structures	SequenceML/RNAStructML
RNAfold [19]	RNA secondary structures folding	SequenceML/RNAStructML

### Workflow systems

More and more bioinformatic tools offered today provide a webservice interface [9-16] in addition to a browser based user interface. In the HOBIT network bioinformatic applications and resources are connected in a uniform way. Webservices are the method of choice for building workflows using bioinformatic tools from different locations.

**Figure 2:**
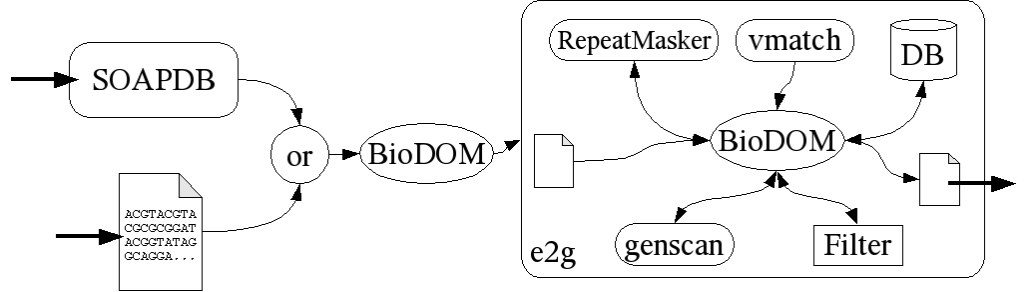
**Graph representation of the e2g workflow**. The e2g webservice gets sequence information in SequenceML format (besides a couple of parameters) as input and returns the result data as an EBIApplicationResult XML document. The input data can originate from a file (containing sequence information as SequenceML) or from an external data source like the SOAPDB webservice (which returns sequence information in FASTA format). Using BioDOM as a converter between the different data formats, it is quite easy to add another data source. The e2g webservice is a workflow itself and also uses webservice technology to mask repeats (using the RepeatMasker webservice) and match the input sequence data against huge EST databases (using the vmatch webservice). The match result is filtered (depending on input parameters) and returned as an EBIApplicationResult document.

Until now connecting webservices is mostly a problem of incompatible data formats which require more or less complex data conversion. The BioDOM library remedies this problem by allowing easy conversion of ordinary formats to HOBIT XML formats. Using standardized input and output formats simplifies combining webservices to a pipeline, thus one webservice choreography (composed of different tasks) could easily be used multiple times without any user interaction. An example is a simple pipeline of SOAPDB offered by DKFZ-Heidelberg and e2g [52] offered by BiBiServ (see figure 2). A sequence is retrieved from the EMBL database via SOAPDB. This sequence is transformed to SequenceML via BioDOM and pushed to the e2g webservice, which maps it to the genomic sequence of the specified species. The result is presented as alignments corresponding to the EBIApplicationResult schema, which is also used by the EBI Blast webservice [10]. Figure 2 shows e2g as an (internal) workflow using RepeatMasker [53], vmatch [54,55] and genscan [56].

Current workflow systems normally require additional programming effort to integrate existing applications without webservice interfaces into complex tool chains. There are two possibilities to minimize the neccessary work using BioDOM:

One can either use BioDOM as a local converter of traditional data, or apply it as a wrapper for a webservice based approach to produce verifiable input and output with the additional value gained by the HOBIT formats (for an example of this approach, see [49]).

Furthermore, new bioinformatic applications can integrate BioDOM directly to support the provided XML formats natively. This eliminates most of the additional effort for workflow integration of the tool.

## Conclusion

Using the HOBIT XML schemas and the BioDOM library, it is easy to add XML support to newly created and existing bioinformatic tools, enabling these tools to seamlessly interoperate in workflow scenarios.

## Availability and requirements

The XML schemas of the HOBIT XML formats can be found on the project's website at .

The BioDOM library is freely available for download in the stable and development versions at  and may be included in external programs under the conditions of the Apache Licence 2.0 (BioDOM requires at least Java Version 1.5, which can be downloaded from ).

Table 4 shows the WSDL locations of SOAP based webservices supporting the HOBIT XML schemas described before.

**Table 4:** WSDL location of SOAP based webservices

**Name**	**WSDL location**
CLUSTALW	
DCA	
Dialign	
E2G	
ITS2	
pknotsRG	
realsplice	
REPuter	
RNAshapes	
RNAfold	

This table shows the currently available bioinformatic webservices for large variety of tasks which support the HOBIT XML data formats as input and output.

## List of abbreviations used

HOBIT: Helmholtz Open Bioinformatics Technology – a cooperative project of several german bioinformatic centres at universities and public research institutes for coordination and interconnection of bioinformatic activities

XML: eXtensible Markup Language

WSDL: WebService Description Language

XSL: eXtensible Stylesheet Language

XSLT: XSL Transformations

DOM: Document Object Model

## Authors' contributions

PNS, KS, JK conceived the study. HM provided the initial work. XML schema design and BioDOM implementation by HM, SH, KL, JK, PNS and KS. PNS, KS, JK, KL and SH drafted the manuscript. RG and TD participated in study design and coordination. All authors read and approved the final version of the manuscript.

**Table 2:** Comparison of native formats and their HOBIT XML counterparts

**Sequence formats**		
FASTA	SequenceML	simple sequence information for nucleic and amino acids
GCG	SequenceAnnotationML	sequence information with additional facilities for annotations
STADEN		

**Sequence alignment formats**		

FASTA	AlignmentML	(multiple) alignments for nucleic and amino acids
CLUSTAL		
MSF		

**RNA secondary structure formats**		

mFOLD	RNAStructML	RNA secondary structure information
Vienna style DotBracket		

**RNA Secondary Structure Alignment Formats**		

aligned Vienna style DotBracket	RNAStructAlignmentML	(multiple) alignments of RNA secondary structures

The table shows a comparison of some native bioinformatic file formats (first column) and their HOBIT XML counterparts (second column). These XML formats cover sequence, alignment, RNA secondary structure and RNA secondary structure alignment formats in a form that is independent of any specific program. The usage of the XML formats leads to a significant decrease in the number of necessary file formats.
